# Causal mediation of plasma metabolomics in pancreatitis: A Mendelian randomization study

**DOI:** 10.1097/MD.0000000000042557

**Published:** 2025-06-06

**Authors:** Yuge Gao, Jia Mi

**Affiliations:** a Changchun University of Chinese Medicine, Changchun, Jilin, China; b Affiliated Hospital, Changchun University of Chinese Medicine, Changchun, Jilin, China.

**Keywords:** genome-wide association study (GWAS), mediation, Mendelian randomization, metabolite ratios, metabolites, risk factors

## Abstract

Pancreatitis frequently leads to hospital stay for digestive system disorders and is in high demand for treatment. To identify possible treatment targets, we utilized Mendelian randomization (MR) to investigate the potential causal effects of metabolites on the outcomes of pancreatitis and examined the intermediary roles of risk factors associated with pancreatitis. We gathered GWAS data on 1091 plasma metabolites and 319 metabolite ratios, along with risk factors and phenotypes associated with pancreatitis and its subtypes. Risk factors included H, T2DM, body mass index (BMI), HLP, cholelithiasis, and Inflammatory bowel disease (IBD). Phenotypic outcomes encompassed acute pancreatitis (AP), chronic pancreatitis, alcohol-induced acute pancreatitis, and alcohol-induced chronic pancreatitis. To test the robustness of the findings, we estimated causality using inverse-variance-weighted MR complemented by sensitivity analyses. Additionally, we performed reverse MR analysis to explore potential reverse causality. This study identified 53 plasma metabolites and 22 metabolite ratios predicted by genetics that were significantly associated with pancreatitis (*P* < .05). Additionally, 54 metabolite ratios and 193 metabolites were associated with pancreatitis risk factors, with 86 and 27 metabolites, respectively, showing significant associations. The MR analysis confirmed that BMI, IBD, and HLP as pancreatitis risk factors (*P < *.05). It was also revealed that BMI and IBD mediate the relationship between certain metabolite levels and pancreatitis. The identified metabolites and their ratios have the potential to serve as circulating biomarkers with promising applications in CP screening and prevention strategies.

## 1. Introduction

Pancreatitis is a multifaceted, advancing, and incapacitating inflammatory condition that affects the pancreas and encompasses clinically diagnosed acute pancreatitis (AP), recurrent AP, and chronic pancreatitis (CP). It is the leading cause of hospitalization for gastrointestinal diseases and is closely associated with higher incidence, mortality rate, and socioeconomic burden.^[1]^ AP has a mortality rate of 1.60 cases per 100,000 person-years (95% CI = 0.85–1.58), while CP has a rate of 9.62 cases per 100,000 person-years (95% CI = 7.86–11.78), with a mortality rate of 0.09 cases per 100,000 person-years (95% CI = 0.02–0.47).^[2]^ Therefore, new and improved strategies for the treatment and prevention of pancreatitis are required. Traditional risk factors, such as alcohol restriction, can prevent nearly 1/5th of cases.^[3,4]^ However, there is a need to identify new risk factors, biomarkers, and treatment methods for pancreatitis. Plasma metabolites^[5]^ are pivotal in biological disruptions commonly associated with illnesses and constitute a significant reservoir for therapeutic targets across a variety of conditions, setting new benchmarks for disease identification. Notably, plasma metabolites are of particular significance in circulatory system disorders, including pancreatitis.^[6,7]^ This is attributed to their direct physical linkage with blood vessels, as opposed to tissue-specific conditions such as ulcerative colitis.

Given that inflammatory conditions of the human pancreas often span a spectrum, AP and CP have many overlapping causes.^[8–10]^ In addition to the overindulgence in alcohol consumption, there are several well-established risk factors for pancreatitis, such as smoking, high triglyceride levels, and autoimmune disorders. Gallstone disease and hypercalcemia can significantly increase the risk of AP, whereas chronic kidney disease and celiac disease are associated with an increased risk of CP. Inflammatory bowel disease (IBD) and systemic lupus erythematosus^[11]^ appear to be associated with a higher risk of pancreatitis, although their exact risk levels are still unknown. Blood markers, including amylase, cholesterol, and C-reactive protein (CRP),^[12]^ are recognized as possible signs of pancreatitis, and observational studies indicate a connection between pancreatitis and metabolic conditions, such as overweight^[13,14]^ and type 2 Diabetes Mellitus (T2DM).^[15,16]^ Although observational research can handle acknowledged confounding factors using statistical methods, the presence of unknown or undocumented confounding factors may still impact the results.

Progress in the genetic domain has substantially aided pancreatitis research via genome-wide association studies (GWASs). GWASs^[17]^ are comprehensive genetic studies that aim to identify genetic variations linked to particular diseases or characteristics. Metabolomics has made it possible to evaluate a multitude of circulating metabolites^[18]^ across extensive population groups, which has facilitated the incorporation of these data into GWASs. Despite this progress, converting genetic findings into an understanding of the biological mechanisms that trigger and advance pancreatitis remains a challenging endeavor. To deepen our understanding of the biological underpinnings of pancreatitis, it is essential to investigate the causal associations between plasma metabolites and their proportions,^[19]^ the probability of developing pancreatitis, and the precise determination of a causal link between blood metabolites and susceptibility to pancreatic diseases, which is hindered by several constraints, including constraints on sample size, the existence of residual confounding elements, and the inherent problem of reverse causality in observational studies. Although randomized clinical trials^[20]^ are considered the gold standard for validating research findings, there are obstacles in assessing the relationship between plasma metabolites and pancreatitis. These obstacles include financial constraints and ethical concerns regarding participant recruitment, which can hinder the conduct of these trials.

Mendelian randomization (MR)^[21]^ has increasingly emerged as a preferred technique for gauging the causal influences of variables on diseases, diminishing biases due to confounding variables or the problem of reverse causation. MR analysis^[22]^ utilizes individual genetic differences, which are randomly assigned at birth, as instrumental variables (IVs). By leveraging comprehensive data from GWAS and associated single nucleotide polymorphisms (SNPs) connected to plasma metabolites, MR analysis establishes a causal relationship between exposure and outcome.

This study aimed to apply a 2-sample MR (TSMR) method to evaluate the causal impact of human plasma metabolites and their ratios on the development of pancreatitis, to ascertain the causal effects of human plasma metabolites and their proportions on the common risk factors that lead to pancreatitis, and to identify plasma metabolites and their ratios that are linked to pancreatitis and its contributing risk factors. Our results provide a foundation for future studies on pancreatitis.

## 2. Materials and methods

### 2.1. Study design

**Figure 1.**
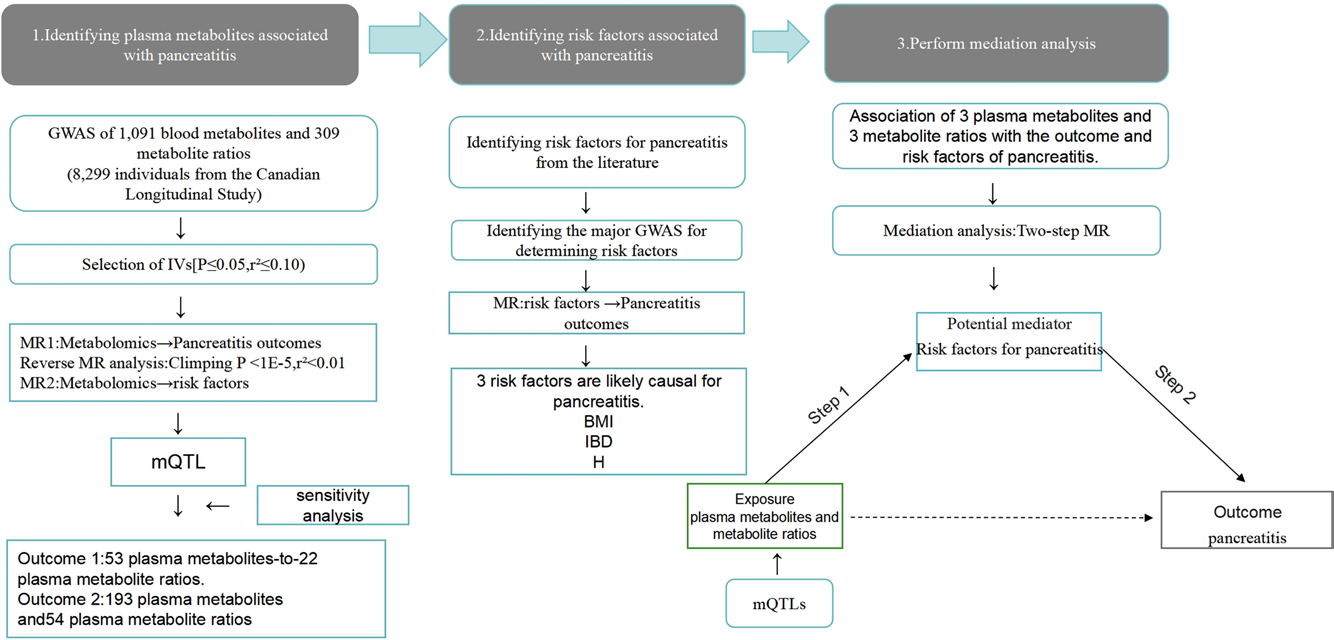
The workflow of MR in this study. MR = Mendelian randomization.

The methodology of this study incorporates magnetic resonance imaging to investigate the causal links between plasma metabolites and their ratios with pancreatitis, and to assess the causal connections between plasma metabolites and ratios with the risk factors that precipitate pancreatitis. Figure 1 provides a summary of the research plan. The MR framework must satisfy 3 essential criteria (Fig. 1): the genetic variants selected as IVs should have a strong correlation with plasma metabolites; the genetic instruments should be disconnected from the pancreatitis outcome, free from possible confounding influences; and genetic differences should be exclusively linked to pancreatitis via plasma metabolites, not through alternative routes.

## 3. Data sources on the lasma metabolites and plasma metabolite ratios

The dataset^[19]^ included genome-wide genotyping data from 8299 individuals in the Canadian Longitudinal Study on Aging cohort^[23]^; 1091 metabolites and 309 metabolite ratios were tested in the GWAS. Among the 1091 plasma metabolites, 850 were known and 241 were unknown. According to the Kyoto Encyclopedia of Genes and Genomes database, recognized metabolites can be categorized into 8 principal metabolic groups: cofactors and vitamins, energy sources, lipids, nucleotides, peptides, amino acids, carbohydrates, and xenobiotics.

## 4. Data sources on pancreatitis and its subtypes

The summary statistics for GWAS used for MR for pancreatitis outcomes were as follows: GWAS summary statistics for AP, CP, AAP, and ACP were sourced from the FinnGen consortium. FinnGen is a significant genomic research project aimed at identifying genotype-phenotype correlations within the Finnish founder population. The FinnGen project’s data gathering comprised 2 elements: approximately 200,000 legacy samples primarily collected by the National Institute for Health and Welfare (THL), and approximately 300,000 prospective samples primarily collected by hospital biobanks. The R10 version of the FinnGen consortium dataset, which encompasses 6787 cases and 361,641 controls pertaining to AP, was used. 3875 cases and 361,641 controls for CP1021 cases and 411,160 controls for AAP and 1959 cases and 410,222 controls for ACP; The data for body mass index (BMI), cholelithiasis, T2DM and hip circumference(H) is sourced from the MRC Integrative Epidemiology Unit, UK. 461,460 controls for BMI; 7682 cases and 455,251 controls for cholelithiasis; 462,117 controls for H; 2972 cases and 459,961 controls for T2DM; The data for IBD is sourced from the International IBD Genetics Consortium.12,882 cases and 21,770 controls for IBD; 394,222 controls for hyperlipidemia (HLP). All study data received ethical clearance from the oversight committee and informed consent was obtained from all participants. Supplementary Data S1, Supplemental Digital Content, https://links.lww.com/MD/O972, displays the characteristics of the summary dataset for pancreatitis and its associated risk factors.

## 5. Risk factors associated with pancreatitis

The main outcomes of this study were the 4 types of pancreatitis. We conducted a PubMed search using terms such as AP, CP, AAP, ACP, and risk factors. Over the past 10 years, we have screened the full texts, guidelines, meta-analyses, reviews, and systematic reviews to identify relevant publications. After this screening phase, we selected several articles for further in-depth analysis. We sought trustworthy, publicly accessible summary statistics from GWAS for these risk factors. The MR analysis included 6 risk factors in 2 samples: BMI,^[24]^ H,^[25]^ T2DM,^[26]^ HLP, IBD,^[27]^ and cholelithiasis.^[28]^ For both secondary and main outcomes, we used the same IV based on the mQTL. The SNP effect sizes for all aforementioned risk factors were derived from previously published GWAS studies (detailed information on the data sources and sample sizes of these GWAS can be found in Supplementary Data S1, Supplemental Digital Content, https://links.lww.com/MD/O972).

## 6. Instrumental variables selection

Herein, we selected SNPs^[29]^ with p-values below the locus-wide significance level (1 × 10^–5^) in the initial analysis as IVs to obtain comprehensive results and enhance the sensitivity to IVs. Subsequently, all IVs underwent linkage disequilibrium clumping (*r*^2^ = 0.001; distance = 10,000 kb) to mitigate the influence of correlated SNPs. Furthermore, Phenoscanner was used to detect possible pleiotropic effects. SNPs exceeding an *F*-statistic threshold of 10 were selected for the subsequent MR analysis because they offered a robust assessment of genetic variation. Ultimately, we omitted palindromic SNPs (in which the active allele was indeterminate) from our study.

## 7. MR analysis

We employed TSMR to gauge the relationships between plasma metabolites and their ratios and outcomes of interest, including pancreatitis and its risk factors.^[30]^ Using this methodology, we identified the genetic correlations with the risk factors within 1 group and examined the link between these genetic markers and the outcome in a different group. Subsequently, we employed fixed-effects or random-effects inverse-variance-weighted (IVW) methods as the primary MR analysis.^[31]^ The choice between fixed-effects and random-effects IVW methods depended on the presence of heterogeneity and pleiotropy. When there was no evidence of heterogeneity or pleiotropy, the fixed-effects IVW model yielded estimates that were statistically significant. When there was no clear heterogeneity or pleiotropy, we generally preferred the random-effects IVW model. The decision to use the random-effects IVW method was based on its ability to provide unbiased estimates by considering potential horizontal pleiotropy and achieving balance in such situations. To bolster the reliability of our findings, we performed sensitivity analyses employing the MR-Egger method, weighted median approach, and MR pleiotropy residual sum and outlier (MR-PRESSO) examination. The MR-Egger method considers the directional influence of the horizontal pleiotropy. If the intercept term significantly differed from zero, it signified the existence of ineffective instruments and suggested a possible bias in the IVW method. The *I*^2^ statistic and *Q*-test were used to evaluate potential heterogeneity and detect outliers in both IVW and MR-Egger analyses. Weighted median analysis indicated that at least half of the instruments were legitimate, and a conclusive overall MR estimate was ascertained by calculating the median of the causal estimates for each SNP. Additionally, the MR-PRESSO test was performed to detect possible horizontal pleiotropy and to correct for its impact by removing outliers.

Here, a *P*-value <.05 was considered nominally associated. IVW and weighted median analyses were applied to mitigate the occurrence of false positives across various tests. Statistical analysis was performed using R software version 4.3.3. The TwoSampleMR and MR-PRESSO packages were used for the MR analysis.

## 8. Mediation analysis

For plasma metabolites and their ratios that exhibit a causal link with both pancreatitis and its risk factors, we conducted a mediation analysis to assess the impact of measuring plasma metabolites and their ratios on the outcome of pancreatitis through these risk factors. The “overall” impact of the exposure on the outcome encompasses both the “immediate” effect and any “secondary” effects that are facilitated through 1 or more intermediaries. In this study, the overall effect was measured using a conventional univariate MR analysis (primary MR). To dissect the immediate and mediated effects, we used the findings from 2-stage MR and applied the multiplication-of-coefficients approach to calculate the beta coefficient of the mediated effect.^[32]^

## 9. Results

### 9.1. Identification of plasma metabolites and plasma metabolite ratios related to pancreatitis

**Figure 2.**
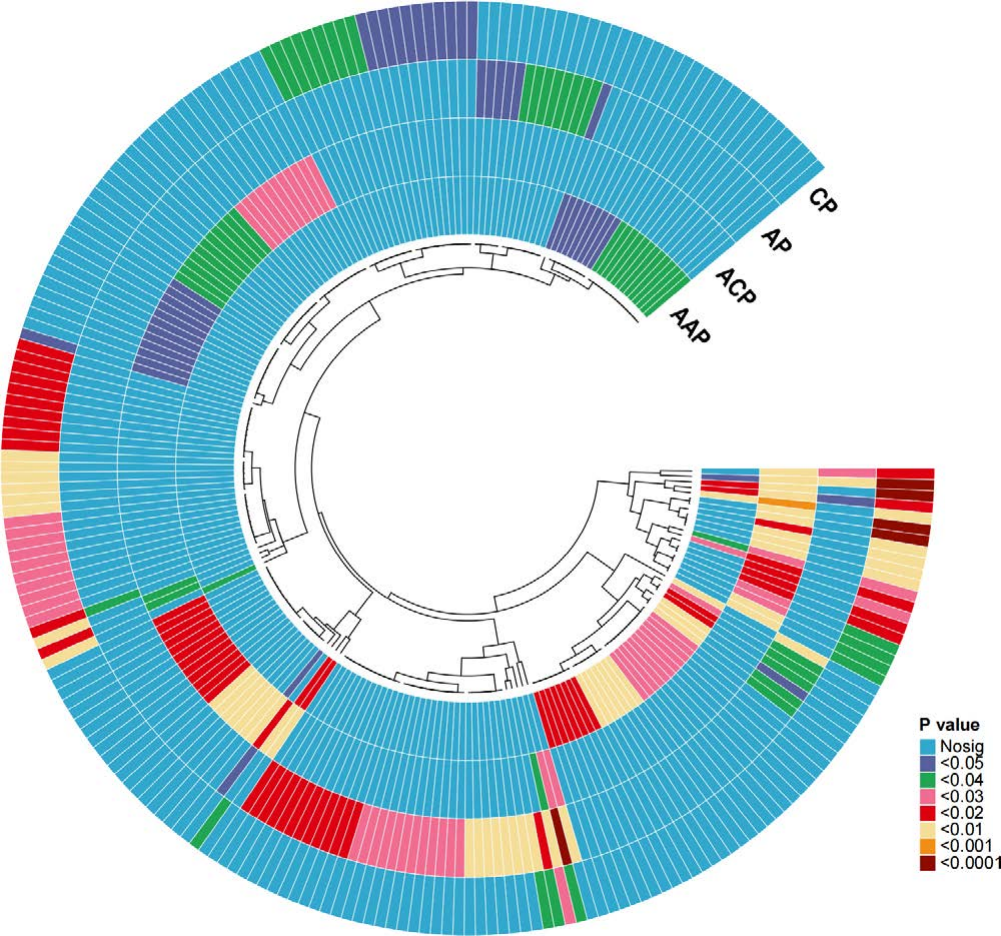
Influence of 53 plasma metabolites and 22 plasma metabolite ratios on pancreatitis outcomes.

A sum of 1091 plasma metabolites and 309 ratios of metabolites were evaluated for their causal link to the outcome of pancreatitis. We initially conducted an MR analysis using mQTLs as IVs and identified 53 plasma metabolites and 22 plasma metabolite ratios that may be associated with at least 1 pancreatitis outcome (*P* < .05). For example, SOGPI (18:0/18:1) levels (OR = 1.126, 9.5% CI = 1.035–1.225, *P* = .006) were associated with ACP, while oleoylcarnitine levels (OR = 1.104, 95% CI = 1.006–1.211, *P* = .037) and PHS (d17:1/16:0) levels (OR = 0.888, 95%CI = 0.83–0.951, *P* = .001) were associated with CP. Additionally, AGPC (20:4n6) levels (OR = 0.949, 95% CI = 0.918–0.982, *P* = .002) were associated with AP (Fig. 2, Supplementary Data S2 and S3, Supplemental Digital Content, https://links.lww.com/MD/O972).

The results of the sensitivity analysis validated the stability of principal MR analysis. No signs of heterogeneity were found in the relationships between the 53 plasma metabolites and the 22 plasma metabolite ratios detailed in Supplementary Data S2, Supplemental Digital Content (https://links.lww.com/MD/O972), as indicated by the Cochran *Q* statistic (PQ-stat > 0.05). Additionally, there were no signs of horizontal pleiotropy based on the MR-Egger intercept (peg-intercept > 0.05) or the MR-PRESSO global heterogeneity test (*P*_GlobalTest_ > .05), suggesting that the IVs did not exhibit directional pleiotropy. All MR causal effect estimates adjusted for IV correlation were consistent with the main analysis. Moreover, MR causal estimates employing IVs derived from a collection of reliable variables that exhibited conditional independence and underwent precise mapping, revealed consistent outcomes.

## 10. Reverse MR analysis

Furthermore, inverse MR analysis was performed to investigate the possible causal effects of pancreatitis phenotypes on plasma metabolite levels and their ratios. This analysis adhered to previously outlined methods. After applying multiple adjustments, our study findings indicated that no connections existed between plasma metabolites and plasma metabolite ratios in any form of pancreatitis, encompassing AP, CP, ACP, and AAP. These divergent results, in contrast to our affirmative MR outcomes, imply an absence of reverse causality.

## 11. Identifying potential risk factors for pancreatitis

To investigate the possible causal pathways linking plasma metabolites with pancreatitis, we performed a 2-stage mediation MR analysis, focusing on conventional risk factors for pancreatitis. Initially, we performed a TSMR to delineate the causal connections between the risk factors for pancreatitis and all outcomes associated with pancreatitis. Subsequently, we assessed the causal impact of metabolites and their ratios on key risk factors.

For each of the 6 risk factors for pancreatitis that we considered, namely hip H, HLP, T2DM, gallstones, BMI, and IBD, IVs were derived from published GWAS summary statistics limited to European populations (Supplementary Data S1, Supplemental Digital Content, https://links.lww.com/MD/O972). Specifically, increases in H, BMI, and IBD increased the risk of pancreatitis (*P* < .05). As expected, the most significant impact was observed for hip circumference on AAP, with an odds ratio (OR) (95% confidence interval [CI], 0.774 [0.659–0.909]). BMI was positively correlated with CP and ACP, with ORs [95% CI] of 1.098 [1.015–1.188]; *P* = .019 and 1.249 (1.176–1.327), *P* = 6.10E-13, respectively. A positive correlation was also observed between IBD and AP, with an OR [95% CI] of (1.037 [1.013–1.061]; *P* = .0019). No association was observed between T2DM, HLP, gallstones, and any outcome of pancreatitis (*P* > .05) (Supplementary Data S4 and S5, Supplemental Digital Content, https://links.lww.com/MD/O972).

## 12. Identification of plasma metabolites and metabolite ratios related to the risk factors of pancreatitis

**Figure 3.**
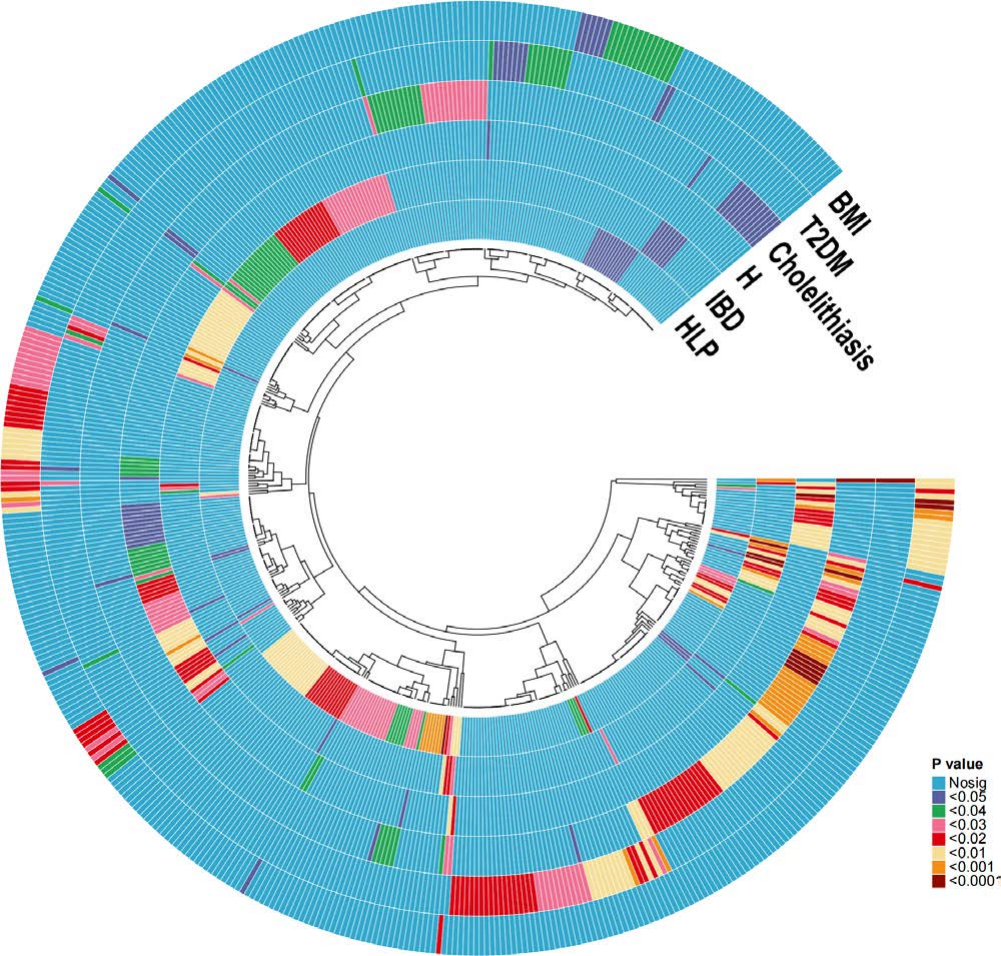
Influence of 86 plasma metabolites and 27 plasma metabolite ratios on risk factors.

We conducted MR analysis on 1091 plasma metabolites and 309 ratios of plasma metabolites, targeting 3 significant risk factors for pancreatitis. Following multiple testing corrections, 86 plasma metabolites and 27 plasma metabolite ratios were found to be significantly associated with at least 1 pancreatitis risk factor (*P* < .05): 32 plasma metabolites and 13 metabolite ratios were associated with IBD, 27 plasma metabolites and 8 metabolite ratios were associated with BMI, and 27 plasma metabolites and 6 metabolite ratios were associated with H. There were no indications of horizontal pleiotropy, and the sensitivity analyses produced uniform estimates of causal impacts (Fig. 3, Supplementary Data S6 and S7, Supplemental Digital Content, https://links.lww.com/MD/O972).

## 13. The mediation effect of plasma metabolites and plasma metabolite ratios on the prognosis of pancreatitis through risk factors

**Figure 4.**
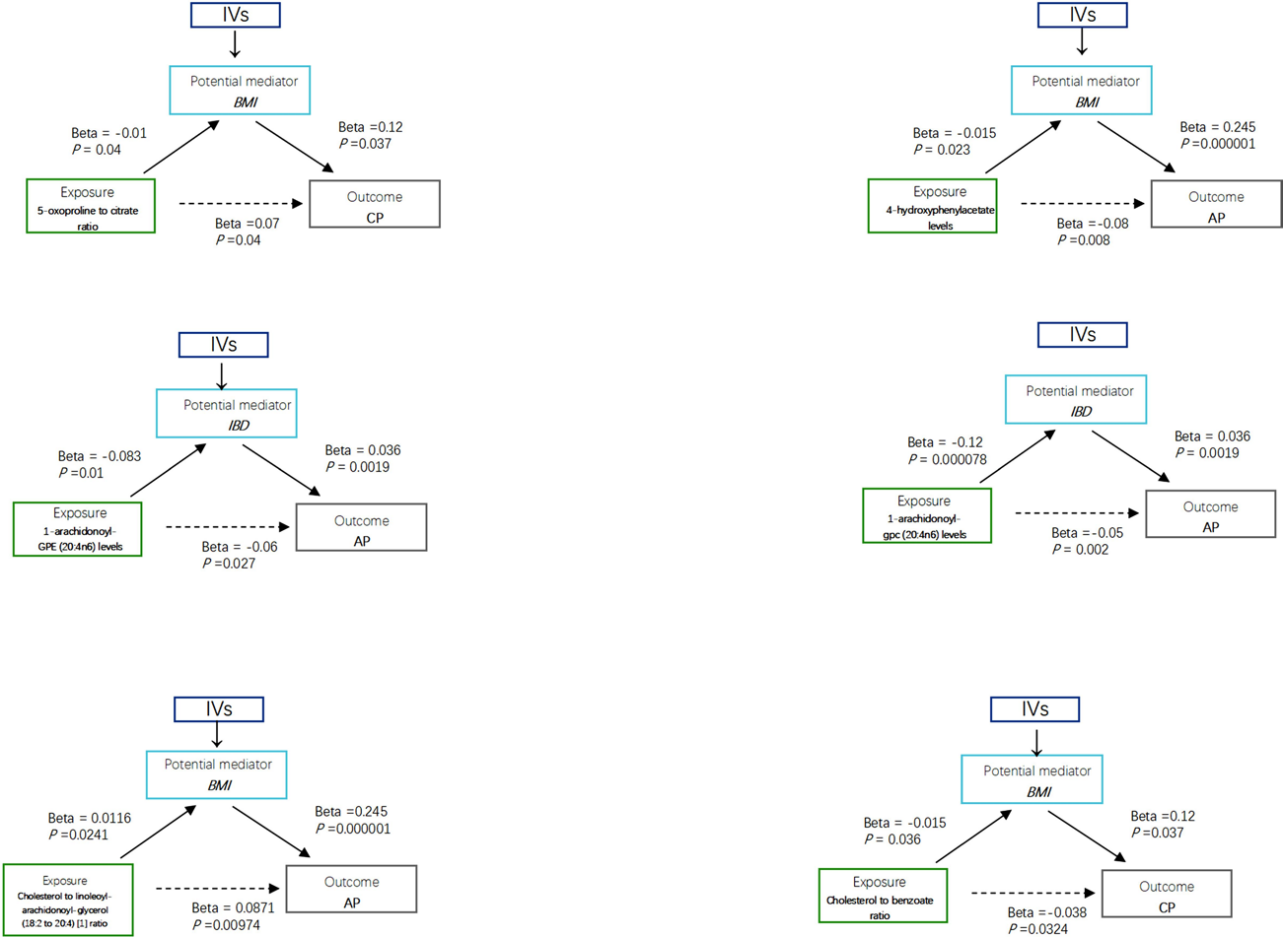
Plasma metabolites and plasma metabolite ratios mediating the effects of risk factors on pancreatitis.

To investigate the mediating effects of plasma metabolites and their ratios on pancreatitis outcomes via risk factors, we performed a mediation analysis utilizing effect estimates from TSMR and the overall effect of a single MR. This analysis was limited to 3 plasma metabolites and 3 plasma metabolite ratios: 4-HPA levels, 1-AGEPE (20:4n6) levels, 1-AGPC (20:4n6) levels, 5-OP to Cit ratio, Chol to LA-AG (18:2–20:4) ratio, and Chol to Ben ratio. To explore the secondary effects of plasma metabolites and their ratios on pancreatitis outcomes mediated by risk factors, we performed a mediation analysis with a 2-step MR for effect estimation and the aggregate effect from a single MR instance. This analysis was limited to 3 plasma metabolites and 3 plasma metabolite ratios: 4-HPA, 1-AGPE (20:4n6), 1-AGPC (20:4n6), 5-OP/Cit ratio, Chol/(18:2–20:4) ratio, and Chol/Benzo ratio. These plasma metabolites and their ratios affected MR analyses of risk factors and stroke outcomes. We calculated indirect effects using the multiplication method and determined the standard error and CI using the delta method. The proportion of the mediating effect of 4-HPA levels on BMI was approximately 4.59% (OR = 0.924, 95% CI = 0.871–0.98, *P* = .008), the mediating effect of 1-AGEPE (20:4n6) levels on IBD was approximately 4.98% (OR = 0.944, 95% CI = 0.897–0.994, *P* = .027), and the mediating effect of 1-AGPC (20:4n6) levels on IBD was approximately 8.64% (OR = 0.949, 95% CI = 0.918–0.982, *P* = .002). The mediating effect proportions through BMI for the 5-OP/Cit ratio and Chol to LA-AG (18:2 to 20:4) ratios were approximately 1.7% (OR = 1.072, 95% CI = 1.004–1.145, *P* = .038) and 4.59% (OR = 1.091, 95% CI = 1.021–1.166, *P* = .009), respectively, and for the Chol to Ben ratio, it was about 4.70% (OR: 0.963, 95% CI = 0.93–0.997, *P* = .032). Among the 54 plasma metabolite ratios and 193 plasma metabolites linked to at least 1 pancreatitis risk factor, 174 metabolite ratios and 43 plasma metabolites were identified as being associated with risk factors, but not correlated with pancreatitis outcomes (Fig. 4, Supplementary Data S8, Supplemental Digital Content, https://links.lww.com/MD/O972).

## 14. Discussion

Our study revealed significant associations between 53 plasma metabolites and 22 plasma metabolite ratios in pancreatitis, thereby providing new insights into the metabolic basis of pancreatitis. Notably, we observed that 1-AGPC (20:4n6) levels were closely associated with the risk of pancreatitis, which may reflect abnormalities in cell membrane metabolism during inflammation. Furthermore, through mediation analysis, we revealed that pancreatitis risk factors such as BMI, IBD, and H may exert their effects by influencing the levels of specific plasma metabolites. These findings provide new strategies for the prevention and treatment of pancreatitis by regulating the levels of these metabolites.

Given the expanding and advancing field of metabolomics research^[33]^ as well as the identification of metabolites associated with pancreatitis, metabolomics will play a crucial role in shaping future stroke treatment strategies. Blood is the most commonly used sample source for metabolomics profiling because it contains a wide range of detectable metabolites and is easily obtainable in large sample sizes, facilitating the screening of circulating biomarkers for pancreatitis risk. Recent metabolomics research has uncovered changes in metabolic pathways and mechanisms at the root of pancreatitis, with lipids, polyamines, and nucleotides frequently being the metabolites highlighted.^[34]^ Our study identified the crucial metabolites associated with the initiation of pancreatitis.

4-Hydroxyphenylacetic acid (4-HPA), an active ingredient found in licorice roots^[35]^ and German chamomiles,^[36]^ has been used in traditional Chinese medicine to treat pulmonary disease.^[37]^ 4-HPA is not only an important polyphenolic compound but also a major metabolite produced through metabolic processes by the intestinal microbiota.^[38]^ It plays a crucial role in antioxidant defense mechanisms,^[39]^ being able scavenging free radicals and reducing oxidative stress-induced cellular damage. Furthermore, 4-HPA reduces the levels of inflammatory cytokines by inhibiting hypoxia-inducible factor-1α (HIF-1α) in NR8383 macrophages, thereby exhibiting anti-inflammatory properties. In the treatment of alcoholic liver disease, 4-HPA has shown potential therapeutic benefits through the modulation of inflammation and lipid metabolism processes^[40]^ 1-Arachidonoyl-2-eicosapentaenoyl-sn-glycero-3-phosphoethanolamine (AEP[20:4n6]) (PE[20:4 (n-6)/20:5 (n-3)]) is a phospholipid compound containing arachidonic acid (AA),^[41]^ and it is vital for cellular communication as well as a range of physiological and pathophysiological functions.

AA is transformed into biologically potent eicosanoids through a range of metabolic routes, including the cyclooxygenase (COX), lipoxygenase (LOX), and cytochrome P450 (CYP450) pathways.^[42]^ These metabolites are essential for modulating vascular performance, inflammatory reactions, immune responses, and various other physiological activities.^[43]^ The irregular metabolism of AA is intimately linked to the emergence and progression of a range of illnesses, including nonalcoholic fatty liver disease (NAFLD), cardiovascular diseases, and inflammatory conditions.^[44]^ Hence, PE(20:4 [n-6]/20:5 [n-3]) and its derivatives have emerged as significant therapeutic targets for various diseases.^[45]^

The gut microbiome modifies the lipid equilibrium of the host by affecting AA metabolism, a mechanism that is vital for sustaining well-being. Additionally, the ratio of 5-oxoproline (5-OPP) to citrate^[46]^ may serve as an indicator for assessing glutamate metabolism and relative activity of the tricarboxylic acid (TCA) cycle.^[47]^ As a precursor of glutathione biosynthesis, the accumulation of 5-OPP is associated with oxidative stress^[48]^ and may serve as a biomarker for oxidative stress. Although the detection method for 5-OPP has not yet been widely used, as research into metabolic and oxidative stress-related diseases advances, the ratio of 5-OPP to citrate might serve as a valuable metric for evaluating disease advancement and therapeutic efficacy.

The ratio of cholesterol-to-linoleoyl-arachidonoyl-glycerol (18:2 to 20:4)^[1]^ reflects the complex relationship between cholesterol^[49]^ and essential fatty acid metabolism. Cholesterol imbalance is associated with the pathogenesis of pancreatitis,^[50]^ and the M6P pathway plays a crucial role in maintaining pancreatic exocrine homeostasis and function.^[51]^ Furthermore, the influence of linoleoyl-arachidonoyl-glycerol (LA-AG) on vitamin E synthesis,^[52]^ as well as its role in cardiovascular diseases^[53]^ and cancer^[54]^ highlights the potential importance of this ratio. Nevertheless, the accurate determination of the cholesterol-to-LA-AG ratio could be quite intricate, necessitating sophisticated lipidomics methods.

The ratio of cholesterol to benzoic acid represents a less-investigated biochemical marker, yet with additional studies, this ratio could reveal its significance in diagnosing and treating diseases.^[55]^ Cholesterol is a vital constituent of cellular membranes and is involved in numerous biochemical processes such as steroid hormone production and bile acid formation. Benzoic acid derivatives can potentially influence cholesterol metabolism indirectly by modulating the metabolic pathways. Dysbiosis of gut microbiota may also affect cholesterol homeostasis. Therefore, further investigation is warranted to clarify the biological relevance and conceivable clinical use of the cholesterol-to-benzoic acid ratio.

Drawing from the outcomes of our study and corroborating them with data from randomized controlled trials, we provide initial prognostic indicators regarding the capacity of these biomarkers in blood assays for the prevention of pancreatitis in subsequent research. This study implies that focusing on particular metabolites may be a fruitful avenue for future studies on the formulation of medications for pancreatitis. This study has several significant advantages. The principal strength of this research was its extensive examination of genetic factors to thoroughly investigate the genetic connections between blood metabolites and the quartet of pancreatitis phenotypes. This study has several significant advantages. Initially, the principal strength of this research was its extensive examination of genetic factors to thoroughly investigate the genetic connections between blood metabolites and the quartet of pancreatitis phenotypes. Third, we leveraged the most extensive dataset accessible for various subtypes of pancreatitis within this domain and carried out thorough sensitivity analyses to validate the reliability of our conclusions.

Nevertheless, this study had certain limitations that warrant recognition. Initially, the study utilized GWAS data specific to exposure and derived outcomes from publicly accessible summary data, where possible sample overlap could lead to confounding bias. Furthermore, distinct data sources might pertain to disparate populations, and such samples might display significant differences in demographic attributes including age, sex, and socioeconomic status. This inconsistency could potentially influence the interpretability of causal estimates and legitimacy of causal deductions. Second, because of the limited number of participants in the exposure dataset, the variety of metabolite types was confined and some linkages between metabolites and pancreatitis might have been detected. Third, the study participants were predominantly of European ancestry and additional research is required to evaluate the applicability of these findings to other demographic groups. Fourth, to bolster the dependability of the research outcomes, we conducted various correction analyses, which could potentially overlook metabolites that have a causal link to pancreatitis. Ultimately, owing to the constrained variance accounted for by SNPs or the limitations of sample size in GWAS findings, some of our MR analyses might not possess adequate strength to identify subtle effects. It is anticipated that future studies using more expansive GWAS datasets will yield enhanced statistical power and more accurate evaluations of genetic influence on metabolites. While the MR approach aids in pinpointing the blood metabolites linked to pancreatitis, forthcoming studies are still essential to delve deeper into their possible mechanisms of action.

## 15. Conclusion

This study systematically investigated the causal relationships and mediating mechanisms between metabolites and pancreatitis and its risk factors by integrating plasma metabolomics with TSMR Among 1091 plasma metabolites and 309 metabolite ratios analyzed, 53 metabolites and 22 metabolite ratios were significantly associated with pancreatitis (*P* < .05). Reverse MR analysis revealed no reverse causal effects of pancreatitis on metabolites (*P* > .05), further supporting the role of metabolic alterations in pancreatitis pathogenesis. Additionally, the study confirmed that BMI and IBD are critical risk factors for pancreatitis (*P* < .05), which indirectly influences disease progression by regulating specific metabolites such as 4-HPA and 1-AGPC. Further analyses identified 86 metabolites and 27 metabolite ratios that were significantly associated with risk factors (BMI, IBD, H, etc) (*P* < .05), while 174 metabolite ratios and 43 metabolites were exclusively linked to risk factors without direct associations with pancreatitis outcomes. This suggests their potential indirect involvement in the pathogenesis of pancreatitis through the modulation of risk factors.

## Acknowledgments

All summary genetic data were obtained from the GWAS project. We thank all participants and investigators for their contribution to the GWAS data.

## Author contributions

**Conceptualization:** Yuge Gao.

**Data curation:** Yuge Gao.

**Methodology:** Jia Mi.

**Project administration:** Jia Mi.

**Software:** Jia Mi.

**Validation:** Yuge Gao.

**Writing – original draft:** Yuge Gao.

**Writing – review & editing:** Jia Mi.

## Supplementary Material


